# South Asia’s unprotected poor: A systematic review of why social protection programs fail to reach their potential

**DOI:** 10.1371/journal.pgph.0002710

**Published:** 2024-06-13

**Authors:** Warda Javed, Zubia Mumtaz

**Affiliations:** School of Public Health, University of Alberta, 3–309 Edmonton Clinic Health Academy, Edmonton, Canada; University of Ghana College of Health Sciences, GHANA

## Abstract

The incongruity between South Asia’s economic growth and extreme poverty has led to a growing interest in social protection and subsequent implementation of anti-poverty initiatives. However, many programs have consistently fallen short of their full potential in reaching the poor. We reviewed the literature to understand the factors behind this failure. A search of EconLit, Global Health Database, MEDLINE and SocINDEX, supplemented by an external search, yielded 42 papers evaluating 23 programs. Inclusion criteria included social and political determinants of program outcomes. Articles were assessed for quality using the GRADE and GRADE CERQual criteria and analyzed using Thomas & Harding’s thematic synthesis approach. We identified five themes: (1) structurally flawed program theories overlook the complexities of poverty and are rooted in simplistic cause-and-effect approaches; (2) elite capture through appropriation of benefits, powerful positioning in program implementation, and gatekeeping through relationships of patronage; (3) insufficient targeting strategies to reach the poorest; (4) neglect of gendered restrictions, hidden costs, lack of legal documentation, and physical and social exclusion; (5) active self-exclusion from social protection to maintain dignity, a perception that programs are substandard, and a lack of resources required. The review highlights the well-documented disconnect between South Asian social protection program designs and the ground realities of their ‘ideal’ beneficiaries. This stems from a dominance of Western-led poverty discourse that disregards the influence of caste, the challenge of effective engagement with a group whose identity remains unclear, and fast-paced funding calls that do not lend themselves to meaningful identification and collaboration with the invisible poor. We suggest this disconnect is intentional and reflects a broader power dynamic rooted in geopolitical interests and national priorities. Study limitations reflect the shortcomings of the existing literature, which largely uses quantitative research methods that fail to capture the multidimensional experiences of the poor.

## Introduction

Over the past few decades, substantial economic growth has been achieved globally, most notably in low and middle-income countries [1,2]. Before the COVID-19 pandemic, the growth was largely accompanied by a concomitant decline in poverty trends [3]. That said, poverty reduction was lower than expected, considering the rate of fiscal expansion. A failure of the trickle-down theory, a lack of pro-poor investment in the social sector, and globalization further left the poor behind and amplified the inequities [1,2,4,5].

This failure to reduce poverty led to a growing interest in social protection, a broad term denoting programs and policies that “[tackle] poverty and vulnerability while strengthening inclusive social development and equitable economic growth.” [6] Usually an *ex-post* response to poverty, the programs intend to establish inclusive growth via promotive, protective [7], preventive [8], and transformative anti-poverty interventions [9]. They include the instruments of social assistance/social safety nets, social care, social insurance, and labour market policies [10]. Through short-term responses to shocks and long-term sustainable and systemic interventions, these strategies aim to support an individual’s movement out of poverty or prevent it altogether [11].

The demand for social protection has heightened due to the multiple overlapping crises of climate change, deglobalization, and, most recently, COVID-19. The pandemic alone has added 124 million individuals [12] to the 648 million [3] already living in extreme poverty, particularly in the world’s resource-poor regions. Social assistance has become the primary tool to cushion these impacts, forming 78% of state-implemented COVID-19 relief [13]. According to recent data, over 1,700 new social protection initiatives were introduced to address the pandemic alone [12].

However, emerging evidence suggests social protection programs fail to realize their potential in targeting the poorest of the poor. A 2022 United Nations report [14] urges that “little attention has been paid to the countless individuals that are falling through the cracks of [social protection].” Kidd & Athias [15] echo these apprehensions in their evaluation of 38 programs across 23 low- and middle-income countries. They show that targeted initiatives did not reach over half the poorest quintile of their anticipated beneficiaries. The Atlas of Social Protection Indicators of Resilience and Equity dataset from 2014–2019 paralleled these findings, showing that social assistance programs in low-income countries covered only 17% of the poorest households [16].

It is particularly important to focus on South Asia, a region home to 21% of the world’s poor [3]. It is plagued with high national debts and inflation [17], corruption, and conflict [18]. South Asia is also on the frontline of the impacts of climate change [19]. Despite the economic progress of some countries, notably India and Bangladesh [20], it remains a region characterized by deeply rooted systemic inequities that subordinate and marginalize some subgroups [21–23]. This marginalization is structured within historical caste-based social hierarchies, which are reinforced by a tangible reality in which the poor have limited rights to engage in the economic, social, and political spheres of their society [24]. An example of this exclusion is the failure of Pakistan’s Benazir Income Support Programme and India’s Mahatma Gandhi National Rural Employment Guarantee Scheme to reach, respectively, 79% and 68% of the poorest quintile of their target populations [15]. Only 25% of Pakistan’s poorest households participate in a social protection program [25].

Social protection is a potentially invaluable tool to address poverty and vulnerability within a world undergoing major changes—whether that be to its climate, AI technologies, or the post-pandemic upheaval. Given the evidence that deserving individuals do not receive their entitled benefits, we reviewed the literature to explore why programs do not reach the poorest of the poor and realize their full potential. As social protection is a vast domain, our review will focus on non-contributory social assistance (e.g., cash transfers, food subsidies, public works programs), microfinance programs, and social health protection. Each of these branches falls within our interests of state-sanctioned and long-term resources targeted toward the poorest of the poor.

## Methods

**Fig 1:**
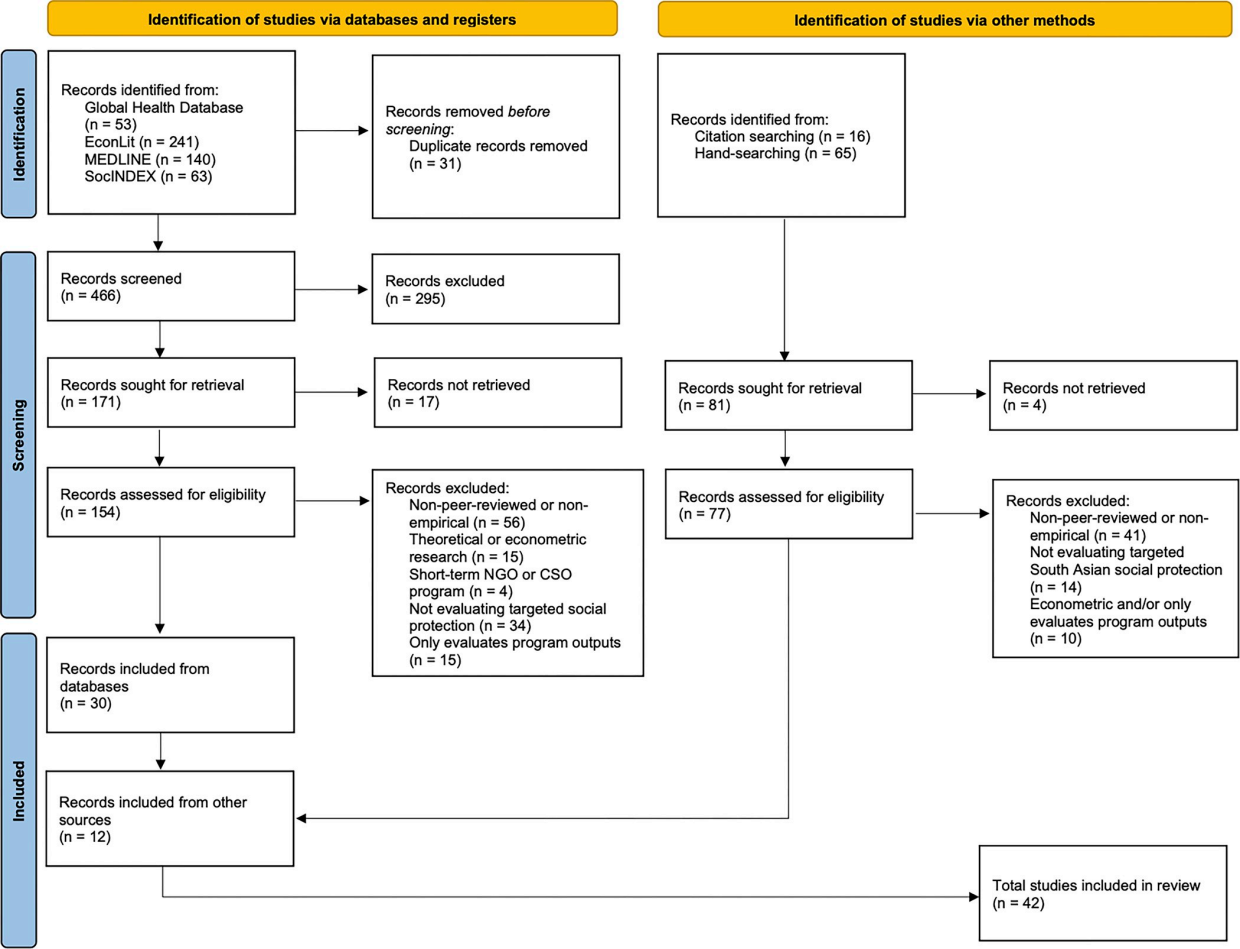
PRISMA 2020 flow diagram.

We conducted a formal search of four databases—EconLit, Global Health Database, MEDLINE, and SocINDEX–which yielded 497 articles. See Table 1 for a list of search terms. These papers were first filtered by title and abstract; 154 were assessed for eligibility based on full-text review, and 30 met our inclusion criteria. Some studies met our inclusion criteria but were excluded as they were short-term donor-funded projects implemented by non-governmental organizations and civil society organizations, or they evaluated program outputs only. We excluded these studies because they were numerous and were characterized by an inherent bias to present a positive result. Other studies were excluded because they evaluated universal programs, not specifically targeted toward the poor. See Table 2 for a full list of the inclusion and exclusion criteria. We supplemented the search through citation tracking from reference sections of our selected papers. Additional supplementary searches about specific programs were performed as needed using Google. This yielded an additional 12 papers. The last search was conducted in September 2023. Fig 1 presents the Preferred Reporting Items for Systematic Reviews and Meta-analysis (PRISMA) [26] flow diagram detailing our search process (S1 and S2 Checklists). Ethics clearance was received from the University of Alberta’s Alberta Research Information Services (ARISE) System (PRO#00117838).

**Table 1:** Keywords in literature search.

“South Asia” OR “India” OR “Pakistan” OR “Bangladesh” OR “Sri Lanka” OR “Afghanistan” OR “Nepal” AND “social protection” OR “programme” OR “safety net” OR “vouchers” OR “income support” AND “poverty” OR “impoverished” OR “vulnerable” OR “socially excluded” OR “caste” OR “marginalized” AND “targeting” OR “efficiency” OR “inequity”

**Table 2:** Inclusion and exclusion criteria for systematic review.

Inclusion Criteria	Exclusion Criteria
Published in the peer-reviewed literature only. English language between 2000–2023. Qualitatively or quantitatively evaluates factors that inhibit South Asian poor from accessing social protection programs. Acknowledges and highlights the role of social and political factors in program outcomes.	Non-English language. Non-peer-reviewed or non-empirical publications (i.e., evaluation reports, working papers, reviews, commentaries, news items, magazines). Theoretical and econometric-driven research. Short-term programs implemented by non-governmental or civil society organizations. Publications not evaluating South Asian social protection programs targeted toward the poor. Evaluation of program outputs only, failing to assess processes related to targeting the poor.

Descriptive data were extracted from each study, including the country of the social protection program, research method, sample size, and key findings. The Grading of Recommendations, Assessment, Development, and Evaluations (GRADE) [27] and GRADE-CERQual (Confidence in the Evidence from Reviews of Qualitative Research) [28] criteria were used to assess the quality of quantitative and qualitative studies, respectively (S1 Table). Mixed-method studies were evaluated using both criteria. Following independent appraisal by the first author (WJ), papers were categorized based on their quality score of high, medium, or low. Only high and medium-quality score papers were included in the review. See Table 3 for detailed summaries of all papers.

**Table 3:** Summary of reviewed literature.

Author, year [reference]	Country	Social Protection Program	Type of Study	Number of Participants/ Secondary Data Source	Significant Findings	Quality of Study
Akerkar *et al*., 2016 [29]	India	Mahatma Gandhi National Rural Employment Guarantee Scheme	Qualitative–in-depth interviews	54 individuals	Social protection dismissed the impact of local moral and political economies in overruling entitlement to social protection.	High
Asadullah & Ara, 2016 [30]	Bangladesh	Targeting the Ultra-Poor	Quantitative–four-round panel survey	Survey 1–5626 households Survey 2–5228 households Survey 3–4559 households Survey 4–4038 households	In short- and medium-term, program participation positively impacts food security, savings, and self-employment. However, the long-term program impact is much smaller.	High
Asri, 2019 [31]	India	National Old Age Pension Scheme	Quantitative–cross-sectional secondary data analysis	India Human Development Survey– 215,753 individuals	Unwarranted possession of the Below Poverty Line ration card and connections to local officials facilitated pension exploitation by non-poor older people.	High
Bechange *et al*., 2021 [32]	Pakistan	Lady Health Workers	Qualitative—in-depth interviews, focus group discussions (FGDs)	73 individuals	Patients did not comply with LHWs referrals for eye care due to gendered mobility restrictions, no transportation, poverty, and a lack of trust in public health care.	High
Das *et al*., 2012 [33]	India	Mahatma Gandhi National Rural Employment Guarantee Scheme	Quantitative–cross-sectional survey	60 individuals	Program corruption and inefficiency resulted from the beneficiaries’ lack of awareness of program guidelines and entitlements.	High
Das, 2015 [34]	India	Mahatma Gandhi National Rural Employment Guarantee Scheme	Quantitative—secondary data analysis	66^th^ National Sample Survey– 281,327 individuals 68^th^ National Sample Survey– 280,763 individuals	Compared to better-off households, the poor were less likely to receive employment through the Scheme after demanding it.	High
Drucza, 2016 [35]	Nepal	Dalit Child Grant, Single Women Allowance	Qualitative–in-depth interviews, surveys	48 beneficiaries, 9 local informants, 14 non-beneficiaries	Cash transfers increased perceptions of social inclusion but did not create equal opportunity. Transfer amounts were low, and governing bodies were often corrupt.	High
Gaiha *et al*., 2001 [36]	India	Integrated Rural Development Programme, Jawahar Rozgar Yojana, Rural Public Works	Quantitative—cross-sectional secondary data analysis	43rd National Sample Survey 50th National Sample Survey	Programs encountered elite capture, mainly through the local *Panchayats*. High inclusion and exclusion errors in all programs.	High
Galasso & Ravallion, 2005 [37]	Bangladesh	Food-for- Education	Quantitative–cross-sectional secondary data analysis	Household Expenditure Survey– 3625 households	Improved intra-village targeting was associated with lower land inequality as the poor had greater decision-making entitlement.	High
Gautam & Andersen, 2017 [38]	Nepal	Nepal Food Corporation, Food-for-Work	Mixed-methods–cross-sectional survey + in-depth interviews, FGDs	313 households surveyed 13 interviews, 10 FGDs	Programs maintained intra-village inequalities. High-caste households exploited their political and social connections to receive more benefits from the program, and community projects were incompatible with the needs of the poor.	High
Hasan *et al*., 2022 [39]	Bangladesh	Shasthyo Shuroksha Karmasuchi	Quantitative—cross-sectional survey	806 households	Program awareness coincided with increased use of subsidized healthcare. Non-Below Poverty Line households were more likely to participate.	High
Imai & Sato, 2012 [40]	India	Integrated Rural Development Programme	Quantitative–cross-sectional secondary data analysis	50th National Sample Survey 55th National Sample Survey	Land inequality negatively impacted program availability. Decentralization was associated with elite capture.	High
Jha *et al*., 2009 [41]	India	Mahatma Gandhi National Rural Employment Guarantee Scheme	Quantitative–cross-sectional survey	942 households	In Andhra Pradesh, wage rates, land inequality, and politicization increased program capture.	High
Jha *et al*., 2011 [42]	India	Rural Public Works	Quantitative—cross-sectional secondary data analysis	50th National Sample Survey 61st National Sample Survey	Program experiences capture from the general population, as well as upper wealth quintiles of the Scheduled Castes and Schedules Tribes.	High
Jha *et al*., 2013 [43]	India	Targeted Public Distribution System	Quantitative–cross-sectional survey	1500 households	Real income transfers through the program were contingent on land inequality, economic status, demographic characteristics, and the high transaction costs of participation. Targeting was insufficient as both poor and non-poor receive subsidies.	High
Jha *et al*., 2013 [44]	India	Mahatma Gandhi National Rural Employment Guarantee Scheme	Quantitative—cross-sectional survey	4561 individuals	Average number of days worked was less than half of the maximum days, and wages were below minimum requirement. However, program wage was higher than local agricultural wage, increasing capture.	High
Kabeer *et al*., 2010 [45]	Pakistan	Public Zakat	Qualitative—FGDs, key informant interviews	Number of interviews unspecified.	Zakat did not reach the poorest of the poor and instead went to well-connected households.	Medium
Kannan & Pillai, 2010 [46]	India	Targeted Public Distribution System	Quantitative–cross-sectional survey	1064 households	Insufficient supply-side resources to address food insecurity and accessibility challenges such as distance, time, and discrimination.	High
Khan, 2021 [47]	India	Targeted Public Distribution System	Quantitative—cross-sectional survey	490 individuals	Destitute informal workers did not have Below Poverty Line cards and could not access the program.	High
Mazumdar & Sharma, 2013 [48]	India	Targeted Public Distribution System	Quantitative–cross-sectional survey	2998 households	High targeting errors as multidimensional deprivations were overlooked in beneficiary identification.	High
Misha *et al*., 2019 [49]	Bangladesh	Targeting the Ultra-Poor	Quantitative–four-round panel survey	Survey 1–5,626 households Survey 2–5,228 households Survey 3–4,549 households Survey 4–4,144 households	Program had a short-term positive impact on employment as participants were likely to switch to entrepreneurship. However, results were unsustainable after nine years as most households that started as beggars or maids returned to the baseline occupations.	High
Mishra & Kar, 2017 [50]	India	National Old Age Pension Scheme	Quantitative—cross-sectional survey	200 households	Scheme encountered high inclusion and exclusion errors and a low probability of participation from the bottom wealth quartiles.	High
Mukherjee & Kundu, 2012 [51]	India	Swarnajayanti Gram Swarozgar Yojana	Quantitative–two-round panel survey	2006 survey– 500 individuals 2008 survey– 469 individuals	Microfinance program expected poor households to repay loans. Targeting via Below Poverty Line classification led to inclusion and exclusion errors. No sampled program participants were extremely poor.	High
Mumtaz *et al*., 2014 [52]	Pakistan	Lady Health Workers, Benazir Income Support Program, maternal health programs (general)	Qualitative–in-depth interviews, FGDs, case studies, observations	94 interviews, 11 FGDs, 134 observation sessions, 5 case studies	Interventions did not reach poor, vulnerable women due to their social invisibility, and socioeconomic dependence on the rich upper-caste to access care.	High
Mumtaz *et al*., 2013 [53]	Pakistan	Lady Health Workers	Mixed-method—cross-sectional survey + in-depth interviews, observations	803 individuals surveyed 48 interviews	Lady Health Workers were more likely to visit and assist those within their *biradari* and social geography. They also encountered limited mobility due to gender and caste.	High
Mumtaz *et al*., 2015 [54]	Pakistan	Community Midwife Program	Qualitative–in-depth interviews, FGDs, observations	239 individuals	Program design was built upon assumptions that overlooked the social and gender norms interfering with the Midwives’ ability to establish private practices.	High
Murgai & Zaidi, 2005 [55]	Bangladesh	Food-for- Education	Quantitative—cross-sectional secondary data analysis	2000 Household Income and Expenditure Survey—7440 households	Program decentralization was pro-poor in that it improved beneficiary identification. However, a high percentage of the eligible population was not covered by the program, and elite capture still occurred.	High
Nair, 2011 [56]	India	Targeted Public Distribution System	Quantitative–cross-sectional survey	400 households	Poor beneficiary identification strategies and high exclusion errors reduced program effectiveness. Well-off withdrew from participation due to low program perception.	High
Nandi *et al*., 2013 [57]	India	Rashtriya Swasthya Bima Yojana	Quantitative—cross-sectional secondary data analysis	2007 District Level Household Survey—590 districts RSBY Web Portal Enrollment Data	Districts with low governance and a high share of low-caste population were less likely to participate. Districts with more non-poor households were more likely to participate.	High
Nayak, 2012 [58]	India	Mahatma Gandhi National Rural Employment Guarantee Scheme	Quantitative—cross-sectional survey	262 individuals	Program experienced elite capture; non-tribal Below Poverty Line cardholders were likelier to participate than tribal. Caste, education, and program awareness were all positive predictors of participation.	High
Nichols, 2016 [59]	India	Food and Nutritional Security programs	Qualitative–semi-structured interviews	81 individuals	Programs that exclusively targeted women enforced an additional gendered labour burden and reduced time commitment to existing workloads.	High
Niehaus *et al*., 2013 [60]	India	Below/Above Poverty Line Classification System	Quantitative–cross-sectional survey	14,074 households	The Below Poverty Line system was not found to be a progressive targeting strategy as it had high inclusion and exclusion errors, likely due to low enforceability.	High
Patel *et al*., 2018 [61]	India	Janani Suraksha Yojana, Accredited Social Health Activists	Qualitative—in-depth semi-structured interviews	18 individuals	Scheduled Caste women encountered barriers in accessing maternal healthcare. These included unjustified excess fees, transportation costs, lack of program information, and discrimination.	Medium
Pattenden, 2011 [62]	India	Mahatma Gandhi National Rural Employment Guarantee Scheme	Qualitative–semi-structured interviews	Number of interviews unspecified.	For low-caste, poor female labourers, accessibility to social protection was dependent on autonomy from the dominant class.	High
Pattenden, 2017 [63]	India	Mahatma Gandhi National Rural Employment Guarantee Scheme	Mixed-methods–longitudinal fieldwork + surveys	1200 households surveyed Number of interviews unspecified.	Dominant class possessed the power to determine whether or not program implementation would be pro-poor.	Medium
RamPrakash & Lingam, 2021 [64]	India	Chief Minister’s Comprehensive Health Insurance Scheme	Qualitative—in-depth interviews	63 individuals	Gendered barriers and household, community, and programmatic level exclusion lowered program participation.	High
Randive *et al*., 2014 [65]	India	Janani Suraksha Yojana	Quantitative–cross-sectional secondary data analysis	District Level House Survey, Annual Health Survey I and II, Census of India	Despite cash incentives, there remained high inequality in maternal mortality rates between rich and poor. Male literacy and poverty explained 70% of the inequality in seeking out institutional delivery.	High
Roy, 2021 [66]	India	Mahatma Gandhi National Rural Employment Guarantee Scheme	Qualitative–quasi-structured interviews and fieldwork	Not Specified.	Labouring class accessibility to the Scheme was a by-product of class coalitions and the political will of the elite.	Medium
Sajid *et al*., 2019 [67]	Pakistan	Public Zakat	Quantitative—cross-sectional survey	486 households	Program experienced capture by upper wealth quintiles. Local Zakat committees also distributed funds based on their discretion.	High
Sinha, 2018 [68]	India	Rashtriya Swasthya Bima Yojana	Quantitative—cross-sectional survey	1643 households	Program did not reduce catastrophic health expenditures or increase facility treatment for poor enrolled households.	High
Walker & Matin, 2006 [69]	Bangladesh	Targeting the Ultra-Poor	Mixed-methods—participatory group exercise + questionnaire	60 individuals	For poor beneficiaries, the program was unsustainable and did not cause any change. Most did not know how to use health cards.	Medium
Zaidi *et al*., 2010 [70]	Pakistan	Livelihood Support Cash Grant	Quantitative–cross-sectional survey	2,612 households	Only one in two households receiving the Grant was eligible, and one in two eligible households were excluded.	Medium

Data analysis was guided by Thomas & Harden’s [71] thematic synthesis approach. This method involves identifying patterns, or themes, across the literature that go beyond the analysis in the primary studies. The iterative and data-driven approach allowed us to synthesize a higher-order interpretation of the literature as opposed to simply summarizing the findings. We first read the papers in detail and conducted a line-by-line coding of all the papers included in the review. The codes were classified to develop descriptive categories. We then conducted axial coding and inductive reasoning to develop broader analytical themes across the 42 papers. Importantly, this is not an exhaustive review but rather an analytical and thematic interpretation of the narrative surrounding South Asian social protection programs.

## Results

The articles in our review evaluate social protection initiatives across four countries—Bangladesh, India, Nepal, and Pakistan—collectively comprising over 95% of the South Asian population [72]. We identified 27 studies from India, seven from Pakistan, six from Bangladesh, and two from Nepal. Twenty-seven studies drew upon quantitative data, 11 used qualitative data, and four used both. The 42 papers researched a total of 23 programs. Table 4 summarizes these programs by country.

**Table 4:** Summary of identified social protection programs*.

Country	Program	Type of program	Eligible population	Program ongoing	Program summary	Literature
**Bangladesh**	Challenging the Frontiers of Poverty-Targeting the Ultra-Poor	Microfinance	Households identified as ultra-poor who meet criteria	Yes	The program aims to help participants graduate from poverty by engaging them in sustainable entrepreneurial activities through education, training, and small loans. Launched in 2002, it targets poor, often landless, women earning less than $0.60-$0.70/day.	[30,49,69]
	Food-for-Education	School enrollment & food subsidy	Poor children in public school	Yes**	Introduced in 1993, the program provided a free ration of grains to school children from poor families, to either consume or sell [73]. Households are targeted by community members who are also responsible for the distribution of entitlements (15kg for one child and 20kg for more than one child).	[37,55]
	Shasthyo Shuroksha Karmasuchi	Healthcare subsidy	Below Poverty Line households	Yes	A pilot project aiming to reduce catastrophic out-of-pocket healthcare expenditures for poor households. The program covers inpatient healthcare services for 78 disease conditions at registered health facilities in three *upzilas* (Kalihati, Madhupar, and Ghatail).	[39]
**India**	Chief Minister’s Comprehensive Health Insurance Scheme	Health Insurance	Below Poverty Line households	Yes	The program is a public-private partnership that covers medical and surgical treatment in empanelled hospitals in the state of Tamil Nadu. Eligible households must have an annual income of less than INR 72000, as proved by a ration card and income certificate.	[64]
	Food & Nutritional Security Programs	Food security	N/A	N/A	A basket of programs aimed at addressing malnutrition by providing food rations, with a focus on mothers and children. It includes the Targeted Public Distribution System, Anganwadi Centres, Auxiliary Nurses and Midwives, and Accredited Social Health Activists.	[59]
	Integrated Rural Development Programme	Microfinance	Below Poverty Line households	Yes**	First launched nationally in 1980, the program provides government subsidies and low-interest credit to poor households. It has now been merged with the Swarnajayanti Gram Swarozgar Yojana initiative, a microfinance program.	[36,40]
	Janani Suraksha Yojana	Cash incentive	Pregnant women in states with low institutional delivery	Yes	A cash-incentive to encourage women to give birth in a health facility. Rural women are given $31 and urban women $22.	[61,65]
	Jawahar Rozgar Yojana	Employment	Rural men and women	Yes**	An employment generation program. Projects are determined by village *Gram Panchayat* members, with priority given to the development of rural infrastructure.	[36]
	Mahatma Gandhi National Rural Employment Guarantee Scheme	Employment	Rural men and women	Yes	Introduced in 2005, the Scheme is a flagship initiative based on a constitutional commitment to provide 100 days of public work to rural households at statutory minimum wage. Rural men and women interested in participating apply through their local *Gram Panchayats*. There they are given job cards and can officially request work by filing written applications; if they are not given employment within two weeks, they are entitled to compensation via unemployment allowance. It is also mandatory that 33% of participants be women.	[29,33,34,41,44,58,62,63,66]
	National Old Age Pension Scheme	Pension	Below Poverty Line aged 60+	Yes	Old age beneficiaries receive 200 INR / month till the age of 79, after which they receive 500 INR / month [74].	[31,50]
	Rashtriya Swasthya Bima Yojana	Health insurance	Below Poverty Line households, and other disadvantaged groups	Yes	Introduced in 2008 as a cushion for poor households to avoid falling into poverty from catastrophic health expenditures. For an annual registration fee of 30 INR, the program covers up to 30,000 INR of inpatient treatment for five household members per year.	[57,68]
	Rural Public Works	Employment	Rural men and women	Yes**	Employment generation program intended to develop rural infrastructure and provide work during agricultural slack periods.	[36,42]
	Swarnajayanti Gram Swarozgar Yojana	Microfinance	Below Poverty Line households	Yes	A microcredit initiative that offers loans to support business development for women from Below Poverty Line households. Through either self-selection or the support of local leaders and organizations, participants are allocated into self-help groups where each member must contribute regularly to the outstanding loan. Through progressive lending, borrowers must pass a series of liability tests to become eligible for a greater amount of credit.	[51]
	Targeted Public Distribution System	Food subsidy	Below Poverty Line and Antyodaya Anna Yojana households	Yes	In 1997, the Public Distribution became a targeted scheme that sells subsidized grains, sugar, oil, and kerosene through government-owned Fair Price Shops. The program was restricted to Below Poverty Line and Antyodaya Anna Yojana households, who were entitled to 35kg of food grains per month, with the latter group having an additional subsidy as they are categorized as the poorest of the poor.	[43,46–48,56]
**Nepal**	Dalit Child Grant	Cash transfer	Poor Dalit children under 5 years of age	Yes	Aims to address food insecurity and nutritional deficits by offering Dalit households 200 NPR per month for a maximum of two children under the age of 5 for five years.	[35]
	Food-for-Work	Employment & food subsidy	Food insecure villages	Yes	Operating under the World Food Program, the initiative aims to improve food security while building rural infrastructure, focusing on agricultural development.	[38]
	Nepal Food Corporation	Food subsidy	Food insecure districts	Yes	Introduced by the government in 1974, the Corporation provides subsidized grains to 30 remote and food insecure districts. Households are allocated 5 kg of rice per person per month.	[38]
	Single Women Allowance	Cash transfer	Widows or single women aged 60+	Yes	Acting as a form of social security allowance, the grant provides single women with a monthly transfer of 500 NPR.	[35]
**Pakistan**	Benazir Income Support Programme	Unconditional cash transfer	Ever-married women aged 18+ who meet eligibility criteria	Yes**	Beneficiaries are paid in quarterly installments of 5,000 PKR [75]. Eligibility criteria include being female, monthly income less than PKR 6,000, no family member in government service, and not being a beneficiary of any other program [76]	[52]
	Community Midwife Program	Healthcare services	Rural women	Yes	Adopted in 2006, the midwife program trains girls in skilled birth attendance to provide maternity care in rural villages. Midwives are expected to attract fee-paying clients by establishing a private practice.	[54]
	Lady Health Workers	Healthcare services	Individuals in rural and urban slums	Yes	Community health worker program aimed at providing domiciliary primary healthcare services with a focus on maternal and child care.	[32,52,53]
	Livelihood Support Cash Grant	Cash transfer	Eligible households in earthquake-affected areas	No	Introduced following the 2005 earthquake to provide affected households with 6 monthly installments of 3,000 PKR [77]. Beneficiary households must meet criteria such as severe housing damage, no male members aged 18–60, and/or low-ranking or no government officers.	[70]
	Zakat Program	Unconditional cash transfer	Poor households	Yes	A tax-financed social transfer program based on the Islamic principle of charity. Funds are distributed by Local Zakat Committees, which are also responsible for identifying the poor.	[45,67]

* Summaries are provided according to the program design at the time of the most recent paper included in the review.

** Program has been re-named, merged with another initiative, or modified.

**Fig 2:**
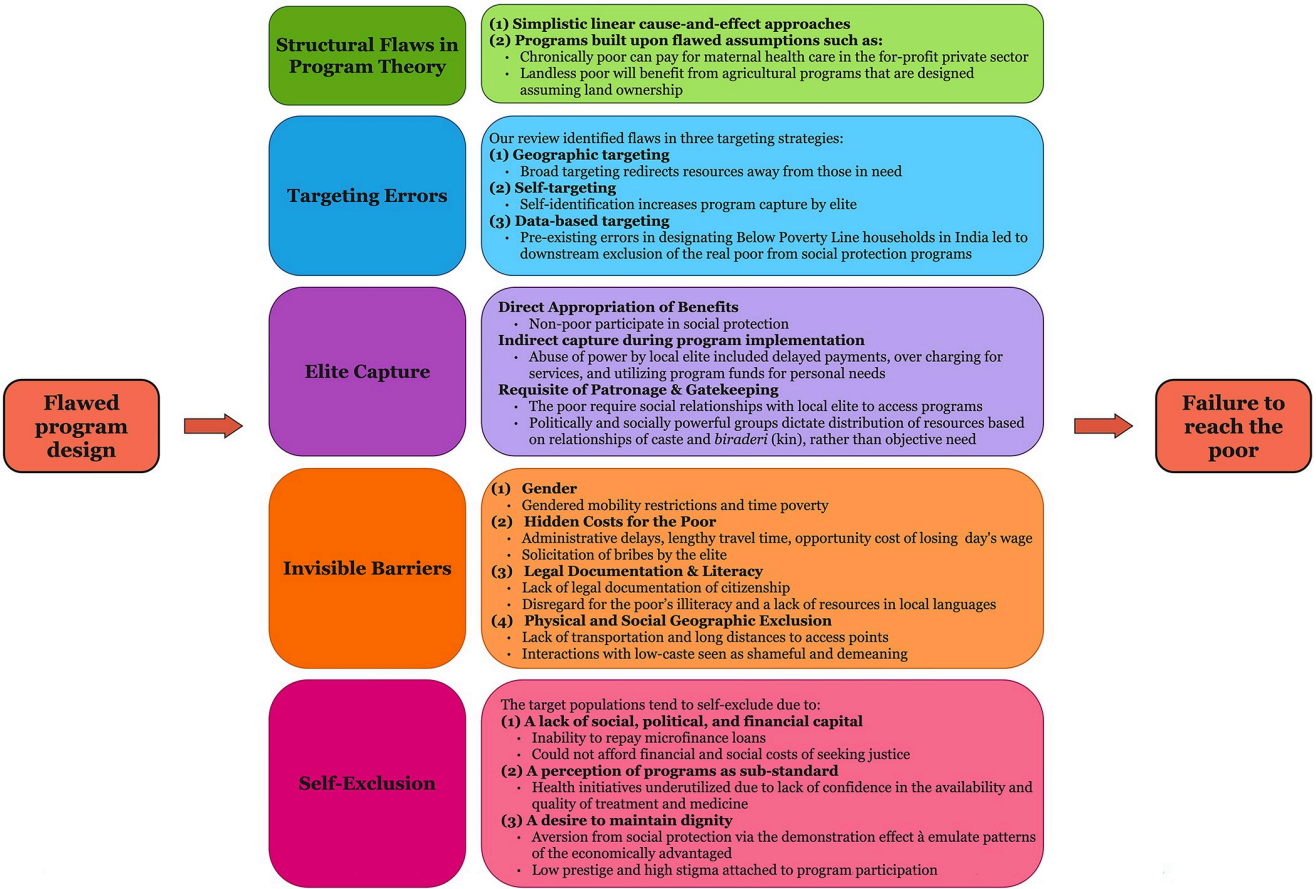
Summary of five themes inhibiting social protection programs from realizing their full potential in reaching the poor.

Our analysis identified five themes underscoring the constructs and processes implicated in withholding social protection programs from reaching the poorest of the poor. These are summarized in Fig 2.

### Structural flaws in program theory

Our review indicates that most programs had flawed program theory, which outlines how an initiative will produce its intended outcomes (i.e., the program design) [78]. Program theories frequently rely on oversimplified linear models of cause and effect and presuppose that their one-dimensional initiatives will produce a chain of consequential impacts. They fail to consider the multi-dimensionality of poverty and how it is rooted in the broader social, economic, and political structures that adversely shape incorporation into society. This is best exemplified by Bangladesh’s Challenging the Frontiers of Poverty Reduction: Targeting the Ultra-Poor initiative, which aimed to redirect the destitute from low-skill occupations through education, income generation training, and microfinance loan services [49,79]. However, evaluations of the program showed that the landless households experienced negligible gains from participation [69]. Entrepreneurial activity increased in the short- and medium-term following program graduation, but the results were not sustainable in the long term [30,49]. Most beneficiaries returned to their baseline occupations, often as beggars and maids [30,49]. We argue this happened because the program’s design assumed that poverty is transient and that a one-time offer of micro-entrepreneurship support was sufficient to matriculate from penury [80]. It ignored the multiple forces operating to keep the poor poor, including their systematic exclusion from markets and the social relationships between and within networks that underpin entrepreneurial activities.

Another example of program theory deficiencies is the Nepal Food Corporation, which provides subsidized rice for the poor. The program required the target beneficiaries to collect the rice from central depots located at significant distances from the remote villages where they live [38]. Since the destitute lack the financial capital to travel to the central depots, the gap in service is filled by individuals with the means to purchase and transport large quantities of grains for the entire village. These middlemen sell the government-subsidized rice for a hefty profit—citing ‘transportation costs’—forcing the poor to purchase their entitled rice quotas through high-interest credit. Consequently, the poor, low-caste Dalits only have 2% of their food needs met through the social protection program [38]. This crucial flaw in program design minimizes the program’s impact on food insecurity, instead nurturing profits for the rich and poverty for the poor.

Other program theories held built-in assumptions that inherently required that beneficiaries have a certain degree of wealth. The Community Midwife Program in Pakistan serves as an illustrative example. This program aimed to ensure skilled birth attendance for impoverished rural women. However, its placement within the private sector proved to be a fundamental flaw [54]. Cognizant of the dissonance between providing a service for the poor and locating the midwives in the private sector, program managers suggested midwives charge a modest fee of USD 0.66 USD for childbirth. Yet, to maintain their practices, the midwives needed to charge fees 10 to 20 times higher than recommended. This private-sector-based approach ultimately failed to meet its objectives, imposing unrealistic financial burdens on both the impoverished women it aimed to serve and the midwives themselves. Over-estimation of the poor’s assets was also evident in Nepal’s Food-for-Work initiative, which has three-fold objectives: (1) improve agricultural output; (2) build road infrastructure to increase market accessibility; (3) provide poor households with employment opportunities by working on the farms or building infrastructure, for which they are paid in-kind with rice [38]. In the district of Humla, the program only benefitted the high-caste, wealthy landowners since they got cheap agricultural labour and road access from their lands to markets. Meanwhile, the landless, chronically poor—the key target group—got nothing more than an insufficient amount of rice, meeting only 15% of a household’s food requirements [38]. The initiative is essentially futile and trivial for the poor, for it failed to achieve its stated objectives of reducing food insecurity and poverty.

### Elite capture of social protection programs

The second theme emerging in our review was the concentration of power in the hands of the South Asian elite and how this impeded program reach to the poorest of the poor. In this context, the denomination of the elite is based on their status as high-caste, with its associated opportunities, privileges, and entitlements [29,38,41,63,66]. This includes land ownership, wealth, and membership in networks that sustain their dominant social and political identities.

#### Direct appropriation of benefits by the elite

The first pattern of elite capture is a traditional form of corruption in which the non-poor participate in welfare programs meant for the poor. For instance, in their sample, Sinha [68] found that 47% of households enrolled in India’s Rashtriya Swasthya Bima Yojana health insurance scheme were non-poor. In Nepal and Bangladesh, the elite captured, or in simpler terms stole, subsidized grains meant for the food-insecure poor. Through secret transactions with program managers in Nepal’s Food Corporation program, large quantities of rice meant for the poor were diverted to local hotel owners to brew alcohol [38]. Similarly, despite providing medium-quality rice to disincentivize wealthy households, the rich still captured Bangladesh’s Food-for-Education program—not for their consumption, but rather as a payment source to their poor servants [37]. The landed rich also captured India’s Mahatma Gandhi National Rural Employment Guarantee Scheme, a program designed to employ the rural poor. Those in the highest wealth quintile not only took over the employment opportunities meant for the landless, the illiterate, and the poor, but they also earned a higher daily wage [29,33,34,44,62,63]. Over 25% of poor households who sought work did not receive it despite being ‘guaranteed’ 100 days of work [34].

#### Elite’s capture of resources through their power over program implementation

The elite also capture resources via the power they hold as social protection program implementers. Most program managers are the local elite since they meet the educational requirements and have the social capital needed to access such job opportunities. This role gives the traditional, informally powerful elite formal power. It simultaneously situates the poor at the mercy of the very people responsible for their subordination. In Nepal, personnel responsible for distributing cash transfers to the poor under the Dalit Child Grant delayed payments, instead redirecting funds into their personal accounts to collect interest [35]. Likewise, the elite captured the implementation of India’s Mahatma Gandhi National Rural Employment Guarantee Scheme. The program aimed to build village infrastructure and simultaneously provide employment opportunities for rural men and women [63,66]. The program planners designated the *Gram Panchayat*, a village-level governing council, as the implementing body responsible for all program tasks, including employment of labourers and allocation of work hours [63,66]. *Gram Panchayat* are traditionally made up of land affluent dominant classes. Many evaluations noted that the *Gram Panchayat* members captured the government-funded labour sources for their personal farms and other private projects [63,66]. For example, Pattenden [63] found that in the village of Panchnagaram, the appropriation of labour led to a third of the local program funding being allocated towards projects on the private lands of the *Gram Panchayat* members. These elites also disincentivized the poor from applying to the program by delaying and withholding wages, sharing misinformation, requiring hidden fees, hiring through nepotism, and, in some cases, preventing registration by withholding applications [29,33,62,63,66]. Ultimately, rather than reducing poverty, the program reproduced and even strengthened the dynamics of traditional inequity [63].

#### The necessity of elite patronage & their gatekeeping role

The literature highlights how the elite often act as gatekeepers, controlling access to social protection for the poor. Here lies the catch-22 of social protection: it is supposed to offer an opportunity for the destitute to lower their dependency and potentially disengage from abusive relationships with the elite, but program design ensures it is precisely these relationships that the poor require to access the programs. Ideally, program accessibility should be based on state-citizen social contracts, but in South Asia, this ideal reigns inferior to the informal contracts with local elites [29,38]. The politically and socially powerful groups dictate the distribution of resources based on caste and *biraderi* (kin) relationships [29,47,54,58,70]. Due to their high decision-making entitlement and low accountability, they act as the poor’s primary route to access social protection [29,64]. In India, these bonds between the powerful and poor are so integral to societal structure that the practice of their formation has been given the special name of *bhav-vyavhar*, or the emotional and behavioural relationship building with the elite [29]. *Bhav-vyavhar* occurs through social transactions such as the poor offering free physical labour, emotional support, and even financial transfers, including a portion of their pension.

In India and Pakistan, some program designs allocated the task of identifying poor households to municipal-level officials and the village elite (often the same group). These officials registered their non-poor family and friends instead of the destitute [56,60]. In India, the poor perceived the National Old Age Pension Scheme as unequal and inaccessible since they lacked the social linkages necessary for participation in the program [31]. This is best reflected in the finding that 74% of deserving recipients were not receiving the Pension [31]. Gatekeeping was also operative in Pakistan’s Zakat initiative, in which Local Zakat Committees, comprised of the community’s well-off, are responsible for identifying the poor and eligible households [45]. An evaluation by Sajid *et al*. [67] showed that in 2019, 40% of the beneficiaries were non-poor.

A powerful tool is the elite’s control of knowledge about the existence and benefits of social programs. Most program designs place the responsibility of disseminating information in the hands of the elite, like the Local Zakat Committees in Pakistan. An evaluation [67] found that the Committees were withholding this knowledge as 80% of eligible beneficiaries in their sample were unaware of the benefits they were entitled to. Das *et al*. [33] also reported censorship of information in their evaluation of India’s Mahatma Gandhi National Rural Employment Guarantee Scheme. The elite forming the *Gram Panchayats* withheld information to ensure local villagers remained unaware of the basic guidelines and operations of the program. Consequently, the poor beneficiaries were underpaid and underemployed.

### Targeting errors

The third theme in our review was the insensitivity of program design when targeting beneficiaries. The literature identified three targeting strategies traditionally used to reach recipients: geographic targeting, self-targeting, and data-based targeting. Geographic targeting purposively selects regions based on poverty or health indicators. Self-targeting (or self-selection) places the onus of identifying eligibility on the poor themselves. Data-based targeting relies on a pre-existing national dataset, such as India’s Below Poverty Line classification. Our review showed that all three mechanisms failed to target their ideal beneficiaries sufficiently and were instead more likely to reach undeserving households.

The Janani Suraksha Yojana program in India illustrates the insensitivity of geographic targeting. This initiative specifically focused on states with low rates of institutional deliveries, introducing a cash incentive scheme to encourage women to deliver in healthcare facilities [65]. An evaluation in nine participating states revealed that all socioeconomic groups experienced a similar average increase in institutional birth. However, the reduction in maternal mortality was four times greater for the richest quintile compared to the poorest [65]. Thus, despite a principal program objective being to lessen financial barriers for *poor* women, the broad geographic targeting mechanism disproportionately benefited the rich living in these localities.

India’s Mahatma Gandhi National Rural Employment Guarantee Scheme serves as a prime example of self-targeting. The program provides employment opportunities for unskilled labourers [44]. An evaluation [44] showed self-targeting permitted uptake by all socioeconomic groups, irrespective of need. In the state of Tamil Nadu, only 40% of the participants were truly poor. Likewise, Pakistan’s Livelihood Support Cash Grant implemented a similarly ineffective form of self-targeting. Applications for the Grant were open to all, but applicants were subject to a screening process based on the government’s predetermined eligibility criteria [70]. Although the program enrolled nearly 700,000 families as beneficiaries, a community-based survey showed that half of the eligible families were excluded, while half of the ineligible rich were provided benefits [70].

A widely recognized static targeting strategy in India is based on classifying households as Below or Above Poverty Line through a state-level census. This classification is not infallible as an evaluation found that 70% of ineligible households in the state of Karnataka possessed Below Poverty Line cards, while 13% of eligible households did not [60]. The downstream consequences of this targeting flaw become evident in various social programs’ failure to reach the poor [48,60]. One example is the failure of Swarnajayanti Gram Swarozgar Yojana microfinance program for the destitute. An evaluation [51] found that none of the beneficiaries in their sample were extremely poor, while over half of the program participants were from high-income households falsely possessing Below Poverty Line cards. Another example is the National Old Age Pension Scheme, in which non-poor Below Poverty Line cardholders were more likely to receive the social allowance than poor cardholders [31]. In fact, Mishra & Kar [50] found that 50% of the Pension recipients were from the top two income quartiles. A third example can be found in India’s Targeted Public Distribution System, a food ration initiative. Five evaluations [43,46–48,56] found that a large fraction of the wrongly classified and wealthy Below Poverty Line cardholders were receiving food rations, while a substantial proportion of the poorest households were not covered by the program. These are worrying findings given that India’s poor lead a dangerous “hand-to-mouth existence” [47].

### Invisible and unacknowledged barriers

Program designs failed to consider a host of barriers handicapping the poor’s accessibility to social protection. These include gendered barriers, hidden costs, the poor’s lack of legal documentation, and the geographies of exclusion. We discuss each below.

#### Gendered barriers

Despite decades of advocacy, programs remain gender blind. In Pakistan, programs like the Lady Health Worker and Community Midwife are specifically designed to deliver domiciliary health care services to home-bound women in direct acknowledgment of the cultural practice of *pardah* (seclusion), and its’ associated value of male honour, limiting women’s unaccompanied movement [53,54,81]. The same gender values also require that the health workers be female. Consequently, only women are recruited as Lady Health Workers and Midwives to provide home-based services. In doing so, the program design paradoxically expects the female health workers to transgress the very gender norms that created the need for their services in the first place. A reading of the papers suggests the program designers were aware of women’s gendered mobility restrictions but chose to ignore the implications of this reality for the female community health workers. This insensitivity and lack of consideration for the young Lady Health Workers and Midwives’ safety and respectability discouraged the workers from assisting households beyond a short walking distance or within their social geography [53,54,81]. The most vulnerable women remained underserved.

Similarly, in Nepal, a food security intervention reproduced gendered inequalities by erring in assuming female labour was a freely available commodity [59]. The program activities added to women’s existing time-consuming socially ascribed domestic duties. This led to female time poverty and subsequent selectivity in program participation [59]. The most effective development strategies were those that relieved gendered labour burdens through services such as the provision of school lunches [59].

#### Hidden costs for the poor

Another crucial structural flaw in program design is the hidden costs required to access the programs. One such cost is the time needed to travel and fulfill the often-onerous bureaucratic requirements, which often result in the loss of a full day’s wage [32,64]. An evaluation of India’s Targeted Public Distribution System in the Sasan sub-district found that 64% of households without ration cards did not even apply out of concern that administrative delays would interfere with work schedules and subsequent earnings [46]. Given the poor’s fragility of income, these opportunity costs can considerably compound the depths of their poverty—resulting in a net financial loss rather than a benefit.

The assumption that time is trivial for the destitute was also exemplified in the design flaws within Pakistan’s Livelihood Support Cash Grants program [70]. To find out if they had been accepted as beneficiaries, poverty-stricken victims of a natural disaster were expected to make regular trips to district offices. If denied, families could only contest their eligibility status within the first seven days following the publication of recipient lists, which were released on an unpredictable schedule. Without regular travel to district offices, poor households could miss the opportunity to appeal—likely a contributing factor to the finding that half of the eligible beneficiaries were not reached by the program [70].

Another hidden cost is the solicitation of bribes. An overwhelmingly common phenomenon within South Asian social protection, corruption is often legitimized under the guise of registration fees and gratuities [29,33,36,47,52,61]. Taking the example of Pakistan’s Zakat cash transfers, the monetary value is reduced as beneficiaries highlight multiple hidden transactional fees from registration officers, peons, and even postmen [45]. In other cases, applicants were completely unaware that they were paying bribes—believing it to be a requisite for program participation [35].

#### Lack of legal documentation and literacy

One common but truly invisible barrier was the poor’s exclusion from documentation of their legal citizenship. Program designs nearly always require such documentation, often an ID card or birth certificate [31,45,47,61,64]. The poor lack these documents for a range of reasons including but not limited to the lack of a permanent address [82], lack of knowledge of the importance of documentation [83], the cost of obtaining documentation [83–85], internal displacement [86], and generational systemic exclusion due to gender [83,84] or caste [85]. These groups contribute to the ‘Invisible Billion’ worldwide who cannot prove their legal identity [84]. In India, the poor’s lack of appropriate documentation meant they could not apply for Below Poverty Line ration cards, which were needed to prove poverty status when registering for the Chief Minister’s Comprehensive Health Insurance Scheme [64]. Some applicants were also excluded from the country’s Rashtriya Swasthya Bima Yojana health insurance as their presented records did not match those of the company handling enrollment [68]. For Pakistan’s Zakat program, these requirements created a black market for illegal birth certificates [45].

Social protection programs also faltered in accommodating the close relationship between poverty and low literacy in rural South Asia [33,70]. Program resources were provided in written format, often in English. The poor’s subsequent inability to absorb the information reified relationships of dependency on the local elite and government staff [33,70].

#### Geography of exclusion

Geographic exclusion encompasses social and physical dimensions, an aspect that social protection programs often overlook in program designs. Despite its seemingly obvious role in accessibility, eight initiatives failed to consider how the lack of transportation and long distances to access points might limit uptake by the poor [32,39,54,61,64,68,69]. The program designs of India’s Targeted Public Distribution System and the Nepal Food Corporation required the destitute to travel to far, often centrally-located, ration shops at their own expense, incurring high transaction costs [38,43,46,56]. In Nepal, Gautam & Andersen [38] found that households with limited mobility resources would spend days walking through harsh terrain to procure their grain rations. Their travel burden was worsened by the uncertainty of whether or not depots would be open or even be capable of meeting their quotas. The program design ultimately penalized the most marginalized, the poor living in remote villages.

Social geography’s less obvious (but critical) role was overlooked in the design of Pakistan’s Lady Health Worker program [53]. Women living in *bhattas* (brick kilns), encompassing the poorest of the poor populations, were unreached by the program because the *bhattas* were considered no-go zones and not a part of human settlement. They were, consequently, beyond the borders of the workers’ catchment areas [52]. There was also the perception that it was “demeaning” to enter *bhattas* [52].

### Gateways to self-exclusion by the poor

Our final theme is the poor choosing to self-exclude from participating in social protection programs. We identified three possible gateways for this process: a desire to maintain dignity, a lack of capital, and a perception of programs as substandard.

The first form of self-exclusion is a desire to maintain a sense of dignity. This is best described by Axel Honneth [87], who says, “We owe our integrity… to the receipt of approval or recognition from other persons.” Nair [56] introduces the idea of aversion to social protection via the demonstration effect in which poor households emulate the patterns of the economically advantaged. This occurred following a transition from a universal to a targeted program in India’s Public Distribution System. As Above Poverty Line households were no longer eligible, low prestige was assigned to those who continued participating in the ration system. Participation stigma was also evident in Pakistan’s Zakat program, complained about the demeaning application process; they felt the government treated them like “beggars” to receive a mere 200 PKR a month [45]. The gravity of aid-affiliated shame is further demonstrated by a village in northern Punjab, Pakistan, in which some poor low-caste women actually preferred to remain invisible and untargeted by social protection to avoid attracting unnecessary attention to their already disgraced identities [52].

The second form of self-exclusion results from the poor lacking the financial, social, and political capital often required to access the resources ostensibly for them. A poverty-stricken household’s awareness of its externally—and sometimes internally—imposed limitations can restrict it from even attempting to access social protection programs. This is evident in the poor’s hesitancy to participate in India’s Swarnajayanti Gram Swarozgar Yojana microfinance initiative due to their fear of being unable to repay their loans [51]. Lack of capital also translated into the sometimes insurmountable challenges of seeking *sunvaiyi* (justice) from government officials [29]. Although the poor had warranted complaints of elite capture, receipt of justice required financial input and high social status to ensure that their grievances would not be disregarded.

The last gateway to self-exclusion results from the perception that programs are of low quality. For example, despite recognizing the benefits of skilled maternal care, Pakistan’s low-caste women continued to seek unskilled birth attendants to avoid experiencing inferior treatment—mitigating the value of government-funded healthcare [52]. In India’s Chief Minister’s Comprehensive Health Insurance Scheme, access to funds took up to 25 days, which delayed treatment even during emergencies [64]. Poor women instead subjected themselves to out-of-pocket payments to receive immediate and poor-quality care. Villagers’ perceptions of programs were also guided by bad experiences of seeking care shared within the community, thus deterring some ideal beneficiaries and fabricating a form of collective exclusion [32].

## Discussion

While our aim is not to belittle the positive outcomes of social protection, the principal conclusion of our literature review is that the root cause of why programs fail to reach the poor lies in program designs that are disconnected from on-the-ground realities. This is not a new discovery. Many authors have commented on this disconnect and have highlighted it as the underlying reason for why programs are not truly transformative [88]. This begs the question, why does this apathy towards the ground realities of the chronically poor persist?

We propose there are three reasons for this. First, there is a lack of understanding of the multidimensional nature of poverty and who precisely the poor are in South Asia. Despite the extensive literature that outlines the caste system’s significant impact on poverty and economic opportunities in the region, it has yet to be integrated into the national-level definitions of poverty and addressed accordingly. This significant oversight is, according to Kabeer [45], a result of the dominance of Western-led neoclassical conceptualizations of poverty developed in Western socio-economic and political cultures in which individuals and households may have the agency to maximise their welfare. She argues that these ideas are inadequate and even inapplicable to South Asian contexts where chronic, intergenerational poverty is anchored in social relationships based on a divinely-presented caste system with its institutionalized processes of exclusion, economic exploitation and political marginalisation of some sub-groups [45].

Second, despite the rhetoric of co-production and end-user engagement [89], there remains a gap in our knowledge of how to precisely engage with the poor [90]. While there’s broad consensus on the principle that co-production should treat every form of knowledge as equal and facilitate open discussions and transformative efforts, the reality of who actually participates in these discussions when researchers and development professionals enter communities is less clear. What are the objectives of community members who engage with outsiders? What is the essence of true community engagement? These questions gain urgency in South Asia where sub-groups of people are stigmatized, discriminated against, or marginalized due to caste identities. They are often rendered invisible to both local elites and visiting professionals [91] and end up not participating in any community engagement activities [92].

Third is the possibility of whether there is even a desire to truly support the poor. The structures and timelines of funding calls do not lend themselves to any meaningful engagement with poor end-users. Despite requiring engagement with ‘local country stakeholders’, donors give 4–6 weeks to develop the program proposals. For example, a recent multi-million-dollar funding call from Global Affairs Canada, ‘Resilient Health Systems for All’ [93], gave six weeks for project design, development of relationships with international stakeholders and write-up. Clearly, there was no time to engage with the primary stakeholders of these projects, the poor. We suggest that the disconnect between program design and poor’s ground reality is intentional and reflects a broader power dynamic. It represents the global elite’s effort to preserve their dominance, with wealthy donor nations and local elites dictating the lives of poorer populations based on geopolitical interests [94] and national priorities [95].

The review had some limitations. The first essentially reflects the limitations of the literature. Twenty-seven of the 42 studies included in our review were quantitative surveys, a rigid data collection method contingent on the quality of questions and their responses [96]. Quantitative results fail to capture behavioural and emotional attributes from respondents, both valuable measures when evaluating the socially and politically driven accessibility barriers encountered by the poor [96]. Second, when identifying papers from outside the databases, we potentially subjected ourselves to the bias of including studies supporting our themes. Third, our review did not capture data from all South Asian countries, as there is a paucity of literature describing and evaluating social protection programs in Afghanistan and Sri Lanka.

Based on this review of the literature, we recommend that the traditional approaches to designing and implementing social protection programs be overhauled and decolonized. This needs to start from the structures of donor funding seeding program ideas and follow-through to national governments that carry the agenda forward. There is a need to critically appraise the international consultants and local decision-makers designing these programs regarding their contextual understanding and accountability. We also recommend more research to understand who the truly poor are and how to best engage them in program design. This requires greater use of qualitative and other innovative research methods. In-depth studies should also be conducted to sociologically evaluate the influence of the caste system on social protection programs, especially in the Muslim-dominant countries of Pakistan and Bangladesh [20,97,98]. The research should contextualize how the power dynamics created by this system are at play in the social, economic, and political spheres and, consequently, must be accounted for in the design of social protection programs. Lastly, research must be conducted to identify how South Asian governments can be held accountable to their poor citizens and effectively absorb the existing knowledge.

## Supporting information

S1 Checklist PRISMA 2020 checklist for systematic reviews. (PDF)

S2 Checklist PRISMA 2020 checklist for abstracts. (PDF)

S1 Table GRADE and GRADE-CERQual quality assessment of included publications. (DOCX)
