# Supplementary material for: South Asia’s unprotected poor: A systematic review of why social protection programs fail to reach their potential
Source: PLOS Glob Public Health. 2024 Jun 13;4(6):e0002710. doi: 10.1371/journal.pgph.0002710 (PMC11175495; doi:10.1371/journal.pgph.0002710)
Supplement: S1 Table — (DOCX) [file pgph.0002710.s003.docx]

**Quality Assessment of Included Literature**

1. GRADE assessment of publications using quantitative research methods

| Study | Question/objective described? | Study design evident? | Method for recruitment subject/comparison groups described? | Subject/comparison groups sufficiently described? | If intervention + random allocation used, described? | Outcome + measures of assessment reported? | Appropriate sample size? | Analysis justified? | Results sufficiently reported? | Conclusion supported by results? | Rating /50 |
| --- | --- | --- | --- | --- | --- | --- | --- | --- | --- | --- | --- |
| Asadullah & Ara (2016) | ✓ | ✓ | ✗ | ✓ | N/A | ✓ | ✓ | ✓ | ✓ | ✓ | 45 |
| Asri, 2019 | ✓ | ✓ | ✓ | ✓ | N/A | ✓ | ✓ | ✓ | ✓ | ✓ | 50 |
| Das *et al.*, 2012 | ✓ | ✓ | ✓ | ✓ | N/A | ✓ | ✗ | ✓ | ✓ | ✓ | 45 |
| Das, 2015 | ✓ | ✓ | ✓ | ✓ | N/A | ✓ | ✓ | ✓ | ✓ | ✓ | 50 |
| Gaiha *et al.*, 2001 | ✓ | ✓ | ✓ | ✓ | N/A | ✓ | ✓ | ✓ | ✓ | ✓ | 50 |
| Galasso & Ravallion, 2005 | ✓ | ✓ | ✓ | ✓ | N/A | ✓ | ✓ | ✓ | ✓ | ✓ | 50 |
| Gautam & Andersen, 2017 | ✓ | ✓ | ✓ | ✓ | N/A | ✓ | ✓ | ✓ | ✓ | ✓ | 50 |
| Hasan *et al.*, 2022 | ✓ | ✓ | ✓ | ✓ | N/A | ✓ | ✓ | ✓ | ✓ | ✓ | 50 |
| Imai & Sato, 2012 | ✓ | ✓ | ✓ | ✓ | N/A | ✓ | ✓ | ✓ | ✓ | ✓ | 50 |
| Jha *et al.*, 2009 | ✓ | ✓ | ✓ | ✓ | N/A | ✓ | ✓ | ✓ | ✓ | ✓ | 50 |
| Jha *et al.*, 2011 | ✓ | ✓ | ✓ | ✓ | N/A | ✓ | ✓ | ✓ | ✓ | ✓ | 50 |
| Jha *et al.*, 2013a | ✓ | ✓ | ✓ | ✓ | N/A | ✓ | ✓ | ✓ | ✓ | ✓ | 50 |
| Jha *et al.*, 2013b | ✓ | ✓ | ✓ | ✓ | N/A | ✓ | ✓ | ✓ | ✓ | ✓ | 50 |
| Kannan & Pillai, 2010 | ✓ | ✓ | ✗ | ✓ | N/A | ✓ | ✓ | ✓ | ✓ | ✓ | 45 |
| Khan, 2021 | ✓ | ✓ | ✗ | ✓ | N/A | ✓ | ✓ | ✓ | ✓ | ✓ | 45 |
| Mazumdar & Sharma, 2013 | ✓ | ✓ | ✗ | ✓ | N/A | ✓ | ✓ | ✓ | ✓ | ✓ | 45 |
| Misha *et al.*, 2019 | ✓ | ✓ | ✗ | ✓ | N/A | ✓ | ✓ | ✓ | ✓ | ✓ | 45 |
| Mishra & Kar, 2015 | ✓ | ✓ | ✓ | ✓ | N/A | ✓ | ✗ | ✓ | ✓ | ✓ | 45 |
| Mukherjee & Kundu, 2012 | ✓ | ✓ | ✓ | ✓ | N/A | ✓ | ✓ | ✓ | ✓ | ✓ | 50 |
| Mumtaz *et al.*, 2013b | ✓ | ✓ | ✓ | ✓ | N/A | ✓ | ✓ | ✓ | ✓ | ✓ | 50 |
| Murgai & Zaidi, 2005 | ✓ | ✓ | ✓ | ✓ | N/A | ✓ | ✓ | ✓ | ✓ | ✓ | 50 |
| Nair, 2011 | ✓ | ✓ | ✓ | ✓ | N/A | ✓ | ✗ | ✓ | ✓ | ✓ | 45 |
| Nandi *et al.*, 2013 | ✓ | ✓ | ✓ | ✓ | N/A | ✓ | ✓ | ✓ | ✓ | ✓ | 50 |
| Nayak, 2012 | ✓ | ✓ | ✓ | ✓ | N/A | ✓ | ✗ | ✓ | ✓ | ✓ | 45 |
| Niehaus *et al.*, 2013 | ✓ | ✓ | ✓ | ✓ | N/A | ✓ | ✓ | ✓ | ✓ | ✓ | 50 |
| Pattenden, 2017 | ✓ | ✓ | ✗ | ✓ | N/A | ✓ | ✗ | ✓ | ✓ | ✓ | 40 |
| Randive *et al.*, 2014 | ✓ | ✓ | ✓ | ✓ | N/A | ✓ | ✓ | ✓ | ✓ | ✓ | 50 |
| Sajid *et al.*, 2019 | ✓ | ✓ | ✓ | ✓ | N/A | ✓ | ✓ | ✓ | ✓ | ✓ | 50 |
| Sinha, 2018 | ✓ | ✓ | ✓ | ✓ | N/A | ✓ | ✓ | ✓ | ✓ | ✓ | 50 |
| Walker & Matin, 2006 | ✓ | ✓ | ✗ | ✗ | N/A | ✓ | ✓ | ✓ | ✗ | ✓ | 35 |
| Zaidi *et al.*, 2010 | ✓ | ✓ | ✓ | ✓ | N/A | ✓ | ✗ | ✗ | ✓ | ✓ | 40 |

45-50 = high quality

30-40 = medium quality

< 30 = low quality

1. GRADE-CERQual assessment of publications using qualitative research methods

| **Summary of review finding** | **Studies contributing to finding** | **Methodological limitations** | **Coherence** | **Adequacy** | **Relevance** | **Confidence** | **Explanation of assessment** |
| --- | --- | --- | --- | --- | --- | --- | --- |
| Bribes in social protection programs | Akerkar *et al.* (2016)  Kabeer *et al.* (2010)  Mumtaz *et al.* (2013a)  Patel *et al.* (2018) | Moderate concerns (one paper with serious concerns, one with moderate concerns, one with minor concerns, but the rest have no concerns) | No or very minor concerns | No or very minor concerns | No or very minor concerns | High confidence | Only concerns are in methodology of three papers. |
| Target population of NREGS provided less than promised 100 days manual labour | Akerkar *et al.* (2016)  Pattenden (2011) | No or very minor concerns | No or very minor concerns | Moderate concerns (only two qualitative studies) | No or very minor concerns | High confidence | Moderate concerns that there are only two qualitative studies supporting the finding (however there are several supporting quantitative studies). |
| Programs delaying payments | Akerkar *et al.* (2016)  Drucza (2016)  Pattenden (2011) | No or very minor concerns | No or very minor concerns | Minor concerns (only three qualitative studies) | No or very minor concerns | High confidence | Minor concerns that there are only three qualitative studies supporting the finding (however there are several supporting quantitative studies). Programs from two different countries. |
| Elite control poor’s access to social protection and distribute resources based on personal relationships. Have strong social and political influence. | Akerkar *et al.* (2016)  Gautam & Andersen (2017)  Kabeer *et al.* (2010)  Mumtaz *et al.* (2014)  RamPrakash & Lingam (2021)  Roy (2021) | Moderate concerns (one paper serious concerns, and two with moderate concerns) | No or very minor concerns | No or very minor concerns | No or very minor concerns | High confidence | Had methodological concerns in some papers but otherwise the finding is very strong and validated. |
| Poor cannot seek justice | Akerkar *et al.* (2016) | No or very minor concerns | No or very minor concerns | Serious concerns (only one study and one interview) | No or very minor concerns | Medium confidence | Although it is only one study, the idea is novel and present indirectly throughout the rest of the literature. |
| Lack of transportation and long distances to access social protection | Bechange *et al.* (2021)  Gautam & Andersen (2017)  Mumtaz *et al.* (2014)  Patel *et al.* (2018)  RamPrakash & Lingam (2021)  Walker & Matin (2006) | No or very minor concerns (one study with minor concerns, one with moderate concerns) | No or very minor concerns | No or very minor concerns | No or very minor concerns | High confidence | Finding was well-researched and discussed throughout interviews from different countries. |
| Losing a day’s wage when seeking social protection | Bechange *et al.* (2021)  RamPrakash & Lingam (2021) | No or very minor concerns | No or very minor concerns | Minor concerns (only two qualitative studies) | No or very minor concerns | High confidence | Minor concerns about adequacy because only two studies, but each from a different country and evaluating different program supporting relevance of finding. |
| Villagers deterred from participating because of community dialogue as programs being of poor quality | Bechange *et al.* (2021) | No or very minor concerns | Minor concerns (potential outliers + not all cases follow this pattern) | Moderate concerns (only one qualitative study) | No or very minor concerns | Medium confidence | Some concerns with data coherence. Concerns about adequacy because only one study. |
| Gender confines women to domestic and reproductive spheres and limits their capability to participate in social protection | Drucza (2016)  Akerkar *et al.* (2016)  Mumtaz *et al.* (2014)  Nichols (2016)  Mumtaz *et al.* (2013b) | No or very minor concerns | Minor concerns (one study highlights that some women from lower castes could go out to work) | No or very minor concerns | No or very minor concerns | High confidence | Only some minor concerns with coherence from one study. |
| Patients were still required to pay a lot of fees in healthcare schemes | Patel *et al.* (2018)  RamPrakash & Lingam (2021) | Minor concerns (one with moderate concerns) | Moderate concerns (programs were still beneficial at reducing OOP) | Minor concerns (only two qualitative studies) | No or very minor concerns | Medium confidence | Concerns about data coherence and methodology. Minor concerns about adequacy because only two studies, but both evaluating different program supporting relevance of finding. |
| Elite responsible for identifying beneficiary households | Kabeer *et al.* (2010)  Mumtaz *et al.* (2013a) | Moderate concerns (one study with serious concerns) | No or very minor concerns | Minor concerns (only two qualitative studies) | No or very minor concerns | Medium confidence | Limitations in methodology and adequacy. However, finding is supported by quantitative results. Moderate concerns about adequacy because only two studies, but each evaluating different program supporting relevance of finding. |
| Failure to adequately publicize social protection registration, information, and guidelines | RamPrakash & Lingam (2021) | No or very minor concerns | No or very minor concerns | Minor concerns (only one qualitative study) | No or very minor concerns | High confidence | Minor concerns about adequacy because only one study (however there are several supporting quantitative studies). |
| Lack of confidence in the quality of treatment in health focused social protection | Mumtaz *et al.* (2013a) | Minor concerns | No or very minor concerns | Minor concerns (only one qualitative study) | No or very minor concerns | Medium confidence | Minor concerns about adequacy because only one study. |
| Stigma associated with program participation | Gaiha *et al.* (2010)  Kabeer *et al.* (2010)  Mumtaz *et al.* (2013a) | Moderate concerns (one with minor concerns, one with serious concerns) | Minor concerns (evidence that suggests beneficiaries would still like to be targeted and participate in program) | Minor concerns (only three qualitative studies) | No or very minor concerns | Medium confidence | Concerns about data coherence and methodology. Minor concerns about adequacy because only three studies, but each evaluating different program supporting relevance of finding. |
| Program overlooked poor’s inability to benefit/participate  Over-estimation of the poor’s benefit from social protection | Gautam & Andersen (2017)  Mumtaz *et al.* (2014) | No or very minor concerns | No or very minor concerns | Minor concerns (only two qualitative studies) | No or very minor concerns | High confidence | Minor concerns about adequacy because only two studies, but each evaluating different program from different country supporting relevance of finding. |
| Lack of legal documentation needed to apply for social protection | Kabeer *et al.* (2010)  Patel *et al.* (2018)  RamPrakash & Lingam (2021) | Moderate concerns (one study with serious concerns, one with moderate concerns) | No or very minor concerns | Minor concerns (only three qualitative studies) | No or very minor concerns | Medium confidence | Moderate concerns with methodology. Minor concerns about adequacy because only three studies, but each evaluating different program supporting relevance of finding. |
| Elite abuse social protection resources and implementation for personal needs | Pattenden (2017)  Roy (2021) | Moderate concerns (one paper with minor concerns and one with moderate concerns) | No or very minor concerns | Moderate concerns (only two qualitative studies) | No or very minor concerns | Medium confidence | Moderate concerns with methodology. Moderate concerns about adequacy because only two studies. |
